# Bimetallic Zeolitic Imidazole Frameworks for Improved Stability and Performance of Intrusion–Extrusion Energy Applications

**DOI:** 10.1021/acs.jpcc.3c04368

**Published:** 2023-09-12

**Authors:** Eder Amayuelas, Sandeep Kumar Sharma, Pranav Utpalla, Jaideep Mor, Luis Bartolomé, Marcus Carter, Benjamin Trump, Andrey Andreevich Yakovenko, Pawel Zajdel, Yaroslav Grosu

**Affiliations:** † Centre for Cooperative Research on Alternative Energies (CIC energiGUNE), Basque Research and Technology Alliance (BRTA), Alava Technology Park, Albert Einstein 48, 01510 Vitoria-Gasteiz, Spain; ‡ Radiochemistry Division, Bhabha Atomic Research Centre, Trombay, Mumbai 400 085, India; § NIST Center for Neutron Research, National Institute of Standards and Technology, Gaithersburg, Maryland 20899, United States; ∥ X-ray Science Division, Advanced Photon Source, Argonne National Laboratory, Argonne, Illinois 60439, United States; ⊥ Institute of Physics, University of Silesia, 75 Pulku Piechoty 1, 41-500 Chorzow, Poland; # Institute of Chemistry, University of Silesia, Szkolna 9, 40-006 Katowice, Poland

## Abstract

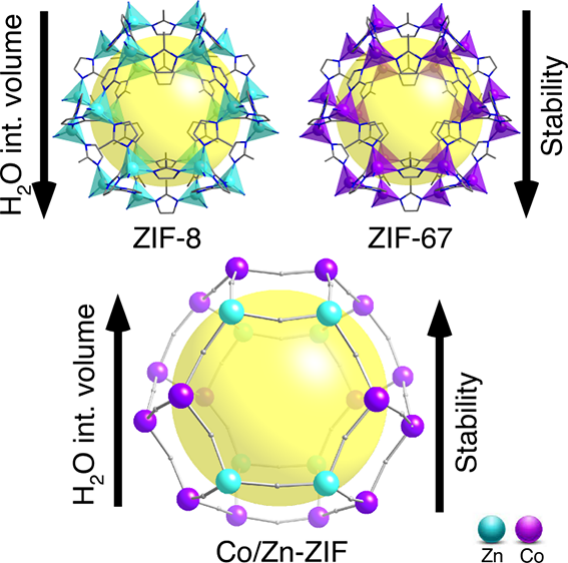

Hydrophobic flexible zeolitic imidazole frameworks (ZIFs) represent reference microporous materials in the area of mechanical energy storage, conversion, and dissipation via non-wetting liquid intrusion-extrusion cycle. However, some of them exhibit drawbacks such as lack of stability, high intrusion pressure, or low intrusion volume that make them non-ideal materials to consider as candidates for real applications. In this work, we face these limitations by exploiting the hybrid ZIF concept. Concretely, a bimetallic SOD-like ZIF consisting of Co and Zn ions was synthesized and compared with Co-ZIF (ZIF-67) and Zn-ZIF (ZIF-8) showing for the first time that the hybrid ZIF combines the good stability of ZIF-8 with the higher water intrusion volume of ZIF-67. Moreover, it is shown that the hybrid-ZIF approach can be used to tune the intrusion/extrusion pressure, which is crucial for technological applications.

## Introduction

Metal–organic frameworks (MOFs) are microporous materials that consist of metal ions or clusters linked through organic ligands, resulting in 3D crystalline structures.^1^ This fact gives rise to a wide diversity of possible combinations between metals and organic linkers,^2^ even the possibility to combine different metallic ions or clusters to enhance physical or chemical properties of these materials.^3^ Thus, MOFs have been studied as potential candidates in areas such as gas adsorption and separation,^4,5^ catalysis,^6,7^ biomedicine,^8,9^ or energy storage^10,11^ among others due to their high surface area, tunable pore size, and diverse chemical functionality. Within these fields, the development of bimetallic MOFs has enabled improving water and thermal stability of benchmark materials in heterogeneous catalysis, luminescence or energy conversion, and storage.^12,13^ Indeed, a facile synthesis and post-synthetic modifications make this sub-class of MOFs even more attractive in terms of tunability to specific applications.

In the last decade, MOFs had attracted great interest in the fields of energy storage,^14,15^ conversion, and dissipation^16−18^ due to their microporous nature with high accessible area and structural diversity. Nevertheless, not many of these materials can withstand the harsh operational conditions of the intrusion-extrusion cycling either due to a lack of hydrophobicity or stability under elevated pressures. To date, only four MOFs were reported to show intrusion-extrusion cycling: ZIF-8,^17^ ZIF-67,^19^ ZIF-71,^16^ and Cu_2_(tebpz).^14^ For this reason, tuning the crucial operational parameters such as intrusion/extrusion pressure is still difficult. In this context, several zeolitic imidazole frameworks (ZIFs) as ZIF-8, ZIF-67, or ZIF-71 arise as reference microporous materials in the community with an increasing number of publications not only related with energy dissipation by means of water intrusion-extrusion^19−21^ but also related with properties and applications derived from this process as nanotriboelectric generators^22^ and giant negative compressibility under hydrostatic pressure.^23,24^

However, these ZIFs, specially ZIF-67, exhibit a lack of stability along several H_2_O intrusion-extrusion cycles due to its instability in water^25^ caused by the fact that Co–N bond that links metal ion to the imidazole is susceptible to hydrolyzation when immersed in water for long periods of time.^26^ This fact makes this material a poor candidate for real operation conditions. In the case of ZIF-8, the stability is much higher as the Zn–N bond is less susceptible to hydrolyzation; however, ZIF-8 exhibits less H_2_O intrusion volume than ZIF-67 and, therefore, lower energy capacity through the H_2_O intrusion-extrusion process.^20,21^

Bearing all this in mind, hybridizing MOFs could broaden this area of study as did for other ones.^27−29^ Turning the perspective from monometallic microporous materials, with individual advantages and disadvantages, to bimetallic materials, that combine those advantages and elude the cited drawbacks, could lead to a new approach of a better rational design of materials as specific solutions to concrete challenges in the area of energy dissipation.

In this work, we synthesized, characterized, and tested Co/Zn hybrid ZIF (Co/Zn-ZIF) as well as its parents ZIF-67 and ZIF-8 and for the first time, subjected it to the H_2_O intrusion-extrusion in order to evaluate differences in the final values as intrusion volume (*V*_int_) and intrusion pressure (*P*_int_) and also their stability carrying out several cycles. Results indicate that bimetallic ZIF solves the cited weaknesses of its counterparts ZIF-67 and ZIF-8.

## Materials and Methods

All chemicals were used as received from reliable commercial sources. Basolite Z1200 (ZIF-8 material) and 2-methylimidazole (HmIm) were purchased from Sigma-Aldrich Co. Cobalt(II) nitrate hexahydrate [Co(NO_3_)_2_·6H_2_O] and methanol 96% were purchased from Fisher Scientific, and ethanol (technical grade) was purchased from Scharlab S.L.

For the synthesis of bimetallic ZIF (Zn_0.6_Co_0.4_) frameworks, 2-methylimidazole (HmIm), zinc nitrate hexahydrate [Zn(NO_3_)_2_·6H_2_O] and cobalt(II) nitrate hexahydrate [Co(NO_3_)_2_·6H_2_O] were procured from Sigma-Aldrich. Analytical grade methanol was procured from J T Baker India.

### ZIF-67 Synthesis

Synthesis of ZIF-67 was based on a method previously reported by Wu et al.^25^ with slight modification. Two separate solutions of Co(NO_3_)_2_·6H_2_O 80 mM and Hmim 320 mM were prepared in 100 mL of methanol. Both solutions were then mixed and stirred for one minute, and the resulting mixture was left for crystallization at room temperature for 48 h. The supernatant was removed, and the purple precipitates were collected by centrifugation and washed thoroughly with ethanol three times. The final product was dried at 80 °C overnight.

### Co/Zn-ZIF Synthesis

The mixed metal ZIF was synthesized following a modified procedure reported by Zhou et al.^30^ using fast current driven synthesis (FCDS). Molar ratio of Co/(Co + Zn) equal to 0.25 was used for the synthesis using the calculated amount of Zn(NO_3_)_2_·6H_2_O and Co(NO_3_)_2_·6H_2_O dissolved in 20 mL of methanol. Zinc and cobalt precursors’ solutions mixed with each other were added slowly to the calculated HmIm solution in methanol connected to the FCDS setup. A small current was passed for 30 min, and after that the solution was left undisturbed for 24 h. A purple-colored precipitate occurred in the solution which was separated using centrifugation and washed with fresh methanol three times. The collected powder was further dried under vacuum (∼10^–3^ mbar) at 100 °C for 6 h to remove the remaining solvent.

### Characterization Techniques

#### X-ray Diffraction

Structural characterization was measured by means of X-ray diffraction at room temperature in powdered samples using a BRUKER-D8 ADVANCE X-ray diffractometer using CuKα (0.15406 nm) recorded in 2θ steps of 0.02° in the 5–80° range.

#### Transmission Electron Microscopy (TEM)

Measurements were carried out by using a FEI Tecnai F20 electron microscope operating at 200 kV. For TEM measurements, samples were placed without any solvent onto a holey carbon film fixed on a 3 mm copper grid.

#### Porosimetry Experiments

H_2_O intrusion-extrusion tests were carried out by means of water porosimetry. Typically, each ZIF material was mixed with water and encapsulated into flexible, hermetic polymeric capsule prior to testing. An Auto Pore IV 9500 porosimeter (Micromeritics Instrument Corporation, Norcross, USA) was used for the compression tests, where the penetrometer was evacuated to a pressure less than 7 Pa, followed by filling with mercury to 50 MPa. For the stability tests, all materials were subjected to 7 H_2_O intrusion-extrusion cycles in the conditions previously described for 24 h. When cycles finished, the materials were taken out from the capsule and dried at 60 °C overnight prior to XRD characterization.

#### Nitrogen Adsorption

Textural characterization was carried out in an automated gas adsorption analyzer (Micromeritics ASAP 2460). Nitrogen physisorption curves were recorded at 77 K after outgassing at 150 °C in vacuum for 15 h. The specific surface area (*S*_BET_) was calculated using the BET equation.^31,32^ For BET equation, we have used the linearization proposed by Parra et al.,^33^ and the pore size distribution was obtained using the Barrett–Joyner–Halenda (BJH) model applied to the desorption isotherm branch. Total pore volume was calculated as the volume of gas adsorbed at a relative pressure of 0.99 in terms of cm^3^/g, using the density of liquid gas to convert the amount of gas adsorbed in STP conditions with the expression *V*_(TOTAL PORES)_ (cm^3^ g^–1^) = *V*_(gas adsorbed)_ (cm^3^ g^–1^, STP) × 0.00154643.

#### FE-SEM

FE-SEM (make: Carl Zeiss, model: GEMINISEM300) was used to capture the morphology of the framework. Co/Zn elemental distribution within the crystal of the frameworks has been determined using energy-dispersive X-ray spectrometry (EDX) coupled with SEM.

## Results and Discussion

**Figure 1:**
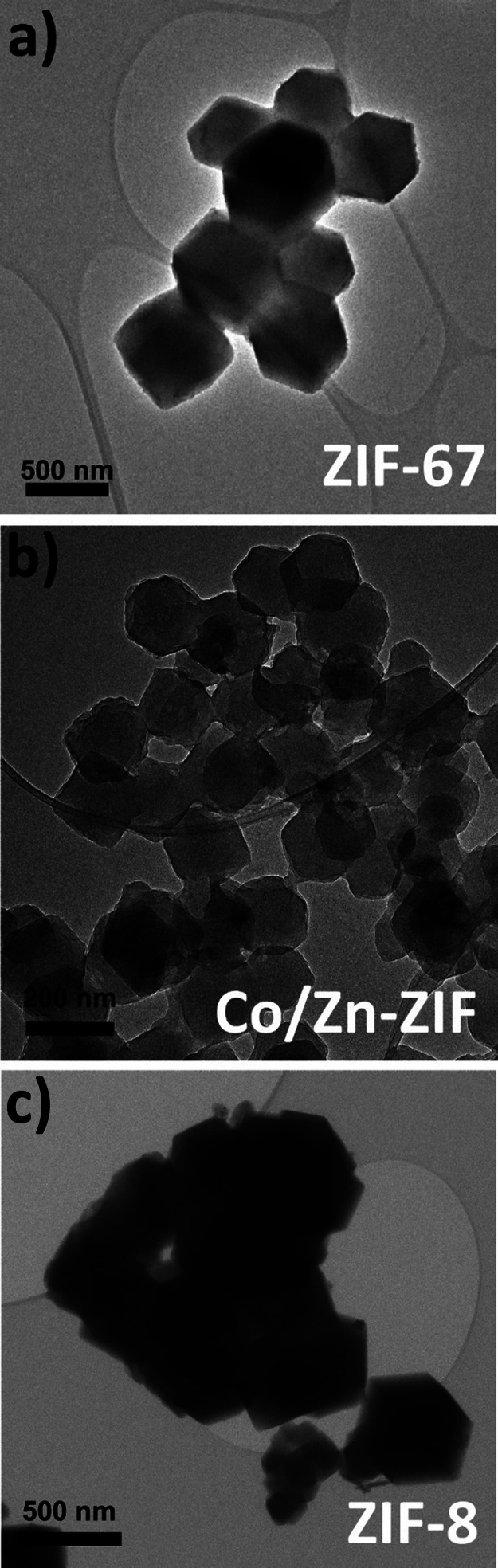
TEM images of pristine (a) ZIF-67, (b) Co/Zn-ZIF, and (c) ZIF-8.

Mainly, commercial ZIF-8 (Basolite Z1200) and synthesized ZIF-67 (synthesis protocol details in Supporting Information) were used as SOD topology reference. Co/Zn-ZIF was synthesized according to the protocol detailed in Supporting Information as a hybrid material of ZIF-8/ZIF-67 based on the combination of Co and Zn ions. As a result, as shown in Figure 1, crystal morphology and shape observed using TEM are similar in the three ZIFs. The three samples are composed of dodecahedral crystals with euhedral morphology and a particle size of ∼500 nm for ZIF-67 and ZIF-8, and ∼200 nm for the hybrid ZIF. These differences in crystal size do not have an impact on the intrusion-extrusion performance as long as the crystal size is above ∼100 nm as was demonstrated in our recent study.^34^ Textural characterization of the three materials was also carried out by means of N_2_ adsorption at 77 K (Figure S1 and Table S1, Supporting Information). Results indicate similar microporous surface area and micropore volume and slightly lower values for ZIF-8.

Therefore, the most significant difference between these three materials lies in their chemical composition, specifically in their metallic cation content, being 100% Zn-based for ZIF-8, 100% Co-based for ZIF-67, and a mixed composition of Co/Zn in the case of the hybrid ZIF. The relative concentration of the two cations present in the structure was characterized by means of EDX coupled with SEM (Figure S2, Supporting Information) confirming the Co and Zn equivalent to the nominal composition in the precursor solutions in the synthesis and resulting in Co_0.4_Zn_0.6_ (Co_2.4_Zn_3.6_ per formula unit, according to ref (30)) despite the fact that Co and Zn are randomly distributed in the framework, as previously reported for this hybrid material.^30^ A simple calculation of crystallographic Z value (formula units per unit cell, eq 1 in Supporting Information) based on previously reported crystallographic information^30^ of this hybrid material gives 2 formula units per unit cell. This means that in each unit cell of Co/Zn-ZIF we have 7.2 Zn atoms per 4.8 Co atoms.

**Figure 2:**
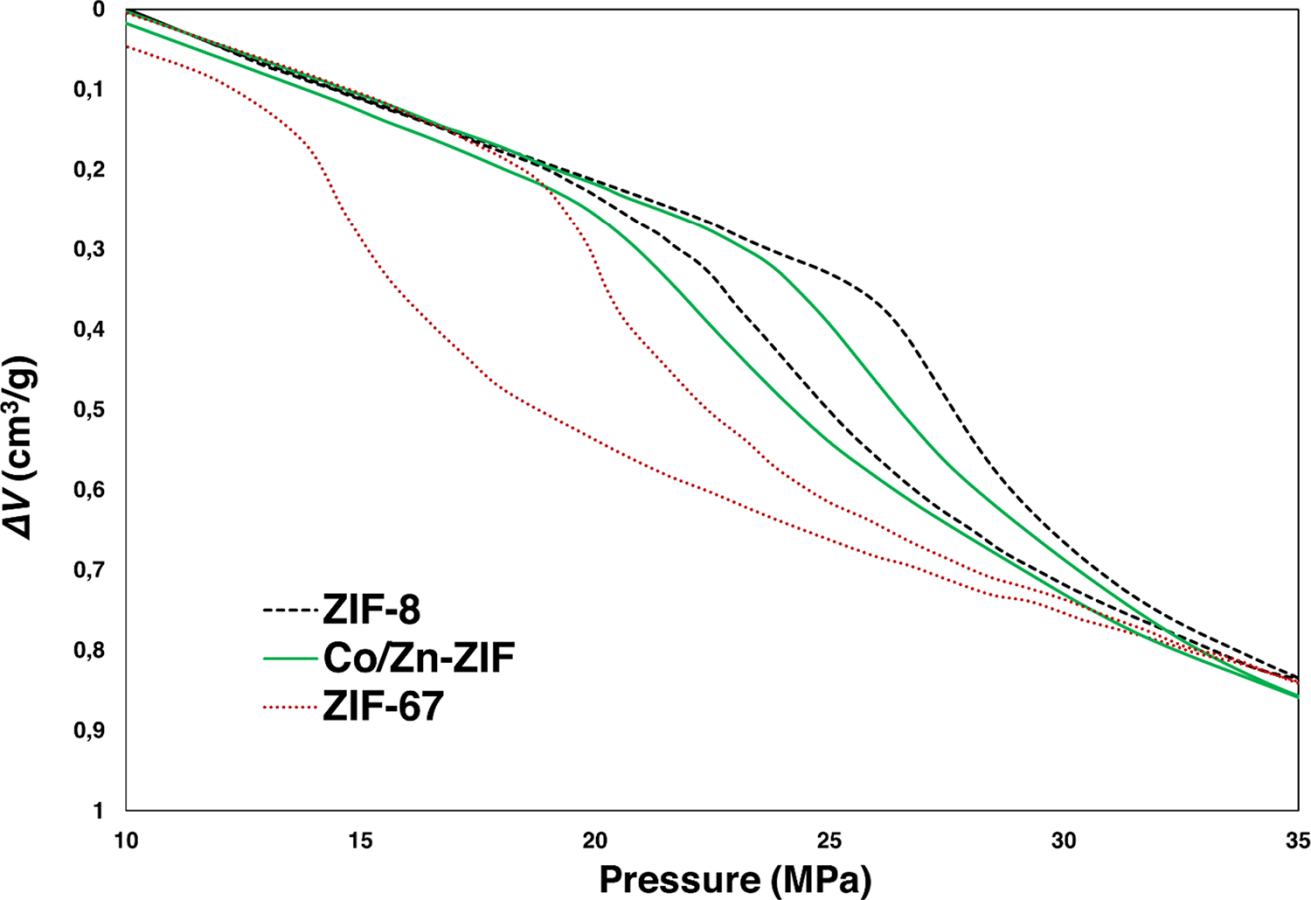
PV-isotherms of ZIF-8 (black dashed line), ZIF-67 (red dotted line), and Co/Zn-ZIF (green line).

The three ZIFs demonstrate clear differences in H_2_O intrusion-extrusion performance (Figure 2). ZIF-67 and ZIF-8 isotherms fit properly with previously reported results,^19−21^ showing relatively high intrusion pressures due to their hydrophobic nature with *P*_int_ values of 20.6 MPa for ZIF-67 and 27.6 MPa for ZIF-8, while in the case of Co/Zn-ZIF, it is 26.0 MPa. The reason for this trend in terms of *P*_int_ can underlie different hypotheses:

1. Structural geometry based on lattice parameters differences between each material.
2. Flexibility of each material during compression stage in the H_2_O intrusion process.
3. Different hydrophobicity of each ZIF.

In order to investigate the mechanism beneath these differences in *P*_int_ and opt for one of the cited hypotheses, a structural analysis of pristine samples was carried out by means of XRD (Table S2 and Figures S3–S11, Supporting Information). Refinement of XRD patterns reveals that lattice parameter is inversely proportional to the intrusion pressure. However, differences in *a* parameter are too small to show such variation of *P*_int_, making the first hypothesis based on geometry very unlikely. Regarding the flexibility case, if we focus on parent ZIF materials’ compressibility (ZIF-67 and ZIF-8) as previously reported measurements in the pre-intrusion stage for ZIF-8, evolution of *a* parameter using synchrotron radiation (data were collected at beamline 17-BM at the Advanced Photon Source, Argonne National Laboratory),^23^ one can confirm that in the case of ZIF-67, the change of lattice parameter is one order of magnitude higher than ZIF-8 (Figures S12 and S13, Supporting Information), which makes water intrude to ZIF-8 at higher *P*_int_, ZIF-67 lower *P*_int_, arising the second hypothesis as the most reasonable so far. On the other hand, a change in hydrophobicity of materials based on different metals could be behind this behavior and would be a path to explore in future deeper studies.

In the case of intruded volume of H_2_O, differences are also clear between ZIF-67, ZIF-8, and hybrid ZIF: 0.34, 0.31, and 0.34 mL/g, respectively. These values reveal that Co/Zn-ZIF present an intermediate behavior between their two parent ZIFs as shown in Figure 2. Hybrid ZIF combines the higher *P*_int_ of ZIF-8 and the higher *V*_int_ of ZIF-67.

**Figure 3:**
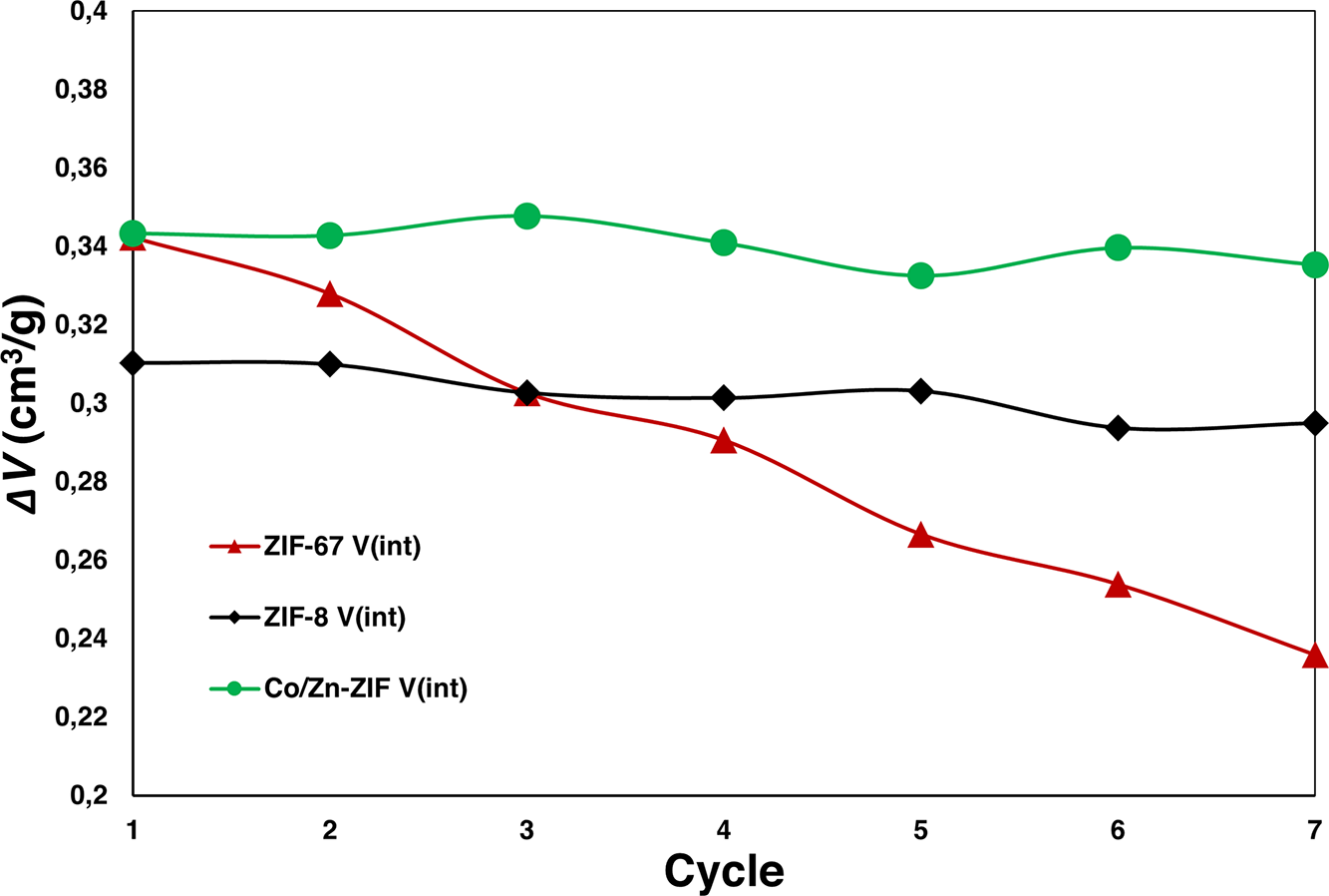
Evolution of *V*_int_ during successive cycles of ZIF-67 (red line), ZIF-8 (black line), and Co/Zn-ZIF (green line).

Subsequently, several H_2_O intrusion-extrusion cycles were carried out to evaluate the stability of the three ZIFs in continuous operation conditions, up to 7 cycles for 24 h. In Figure 3, intrusion volume (directly related with micropore volume and, therefore, with integrity of porous nature of the materials) is plotted versus cycles, showing again clear differences between three materials (Table S3 in Supporting Information). ZIF-67 and Co/Zn-ZIF start from the same initial *V*_int_ which is consistent with the similarities in microporosity values previously mentioned (Figure S1 and Table S1 in Supporting Information); however, ZIF-67 values drop drastically with each cycle up to 65% of its maximum *V*_int_. The hybrid ZIF remains stable during cycles with negligible drop of H_2_O intruded volume as in the case of ZIF-8, well known for its stability throughout the successive intrusion-extrusion cycles.^21,35^

**Figure 4:**
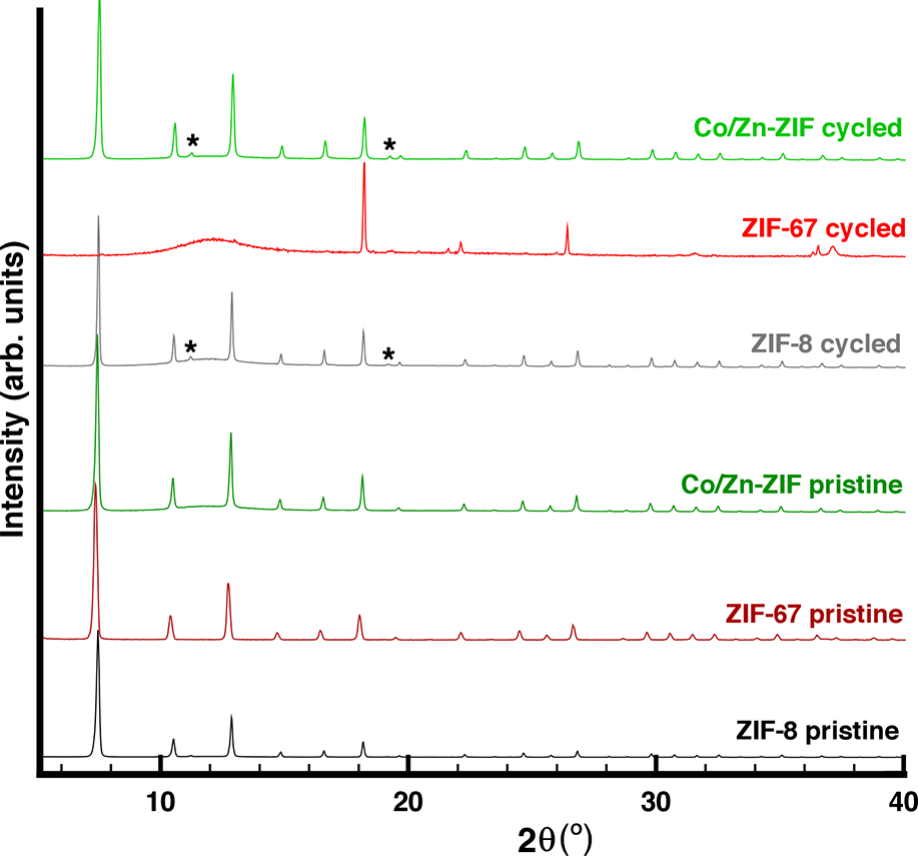
XRD patterns of the pristine ZIF-8, ZIF-67, and Co/Zn-ZIF compared to the patterns of the same samples after 7 H_2_O intrusion-extrusion cycles (* indicates unknown phase).

The study of stability of the three SOD ZIFs was completed by means of powder X-ray diffraction comparing the XRD patterns of the pristine samples and the patterns of the cycled samples. As shown in Figure 4, the patterns of the three pristine samples are indistinguishable as the three ZIFs are isostructural SOD-like materials. However, the XRD pattern of pristine Co/Zn-ZIF sample exhibits a small “bump” in the background between 10° and 15° in 2θ, which is not present in the other two ZIFs materials. The structured background is an indication of a short range order and is common in glassy or polymer materials. This could be indication (but not proof) of an amorphous phase. However, the presented analysis of intrusion volume demonstrates that its contribution is negligible. After cycling, in the case of ZIF-67, the crystal structure undergoes degradation as expected,^25^ while in the case of ZIF-8 and Co/Zn-ZIF, all peaks from the initial patterns remain identical in the cycled ones. However, two small reflections appear at ∼11° and at ∼19° in 2θ, respectively, indicating the presence of an unknown phase. Even if these two extra peaks are related with the formation of new phases from the H_2_O intrusion-extrusion process, they do not affect the performance of these materials.

One can see that this qualitative analysis of XRD patterns confirms the integrity of crystal structure of hybrid ZIF compared to ZIF-67. This is an expected behavior since the main metal ion in the structure of Co/Zn-ZIF is Zn. As previously mentioned, the main proposed mechanism of the degradation of ZIF-67 is the hydrolyzation of Co–N bond when immersed in water for long periods of time,^25^ so degradation of hybrid ZIF would be expected to occur in the same way. However, this process is prevented or, at least, highly minimized, by introducing a determined concentration of Zn cations in its structure. What is the role of Zn in preventing this degradation mechanism is an open question of this study that also opens new topics to dive in for future studies as follows:

the minimum concentration of Zn cations to avoid or minimize degradation.
the impact of different Co:Zn ratios in the hybrid structure to unlock higher *V*_int_ values.
the impact of different Co:Zn ratios in properties resulting from H_2_O intrusion-extrusion under hydrostatic pressure as negative compressibility.

## Conclusions

In summary, a bimetallic ZIF composed of Co and Zn cations was synthesized inspired by an asymmetric mixture of ZIF-67 and ZIF-8 with metallic ion concentration of Co_0.4_Zn_0.6_. The H_2_O intrusion-extrusion tests clearly show that the combination of a slightly higher proportion of Zn ions and a lower proportion of Co ions in its crystal structure results in a hybrid ZIF with a high H_2_O intrusion volume (similar to ZIF-67) and a high stability (similar to ZIF-8).

This study represents the first study with a bimetallic MOF used for intrusion-extrusion application. It represents an interesting starting point for the introduction of bimetallic microporous compounds in the field of energy storage, conversion, and dissipation as well as triboelectrification and negative compressibility with the objective of not only to enhance the performances of benchmark materials in the field but also to expand the knowledge that comes out to light derived from this methodology.

## Supporting Information Available

Details on the synthesis and characterization of all tested materials (PDF)
