# Supplementary material for: Bimetallic Zeolitic Imidazole Frameworks for Improved Stability and Performance of Intrusion–Extrusion Energy Applications
Source: J Phys Chem C Nanomater Interfaces. 2023 Sep 12;127(37):18310–5. doi: 10.1021/acs.jpcc.3c04368 (PMC10518860; doi:10.1021/acs.jpcc.3c04368)
Supplement: Supplementary file 1 — jp3c04368_si_001.pdf [file jp3c04368_si_001.pdf]

Bimetallic Zeolitic Imidazole Frameworks for Improved Stability and Performance of Intrusion-Extrusion Energy Applications

Eder Amayuelas,[1] Sandeep Kumar Sharma,[2] Pranav Utpalla,[2] Jaideep Mor,[2] Luis Bartolomé,[1] Marcus Carter,[3] Benjamin Trump,[3] Andrey Andreevich Yakovenko,[4] Pawel Zajdel,[5] Yaroslav Grosu.[1,6,*]

[1]*Centre for Cooperative Research on Alternative Energies (CIC energiGUNE), Basque Research and Technology Alliance (BRTA), Alava Technology Park, Albert Einstein 48, 01510 Vitoria-Gasteiz, Spain.*

[2]*Radiochemistry Division, Bhabha Atomic Research Centre, Trombay, Mumbai, 400 085 India.*

[3]*NIST Center for Neutron Research, National Institute of Standards and Technology, Gaithersburg, Maryland 20899, USA.*

[4]*X-Ray Science Division, Advanced Photon Source, Argonne National Laboratory, Argonne, Illinois 60439, USA.*

[5]*Institute of Physics, University of Silesia, 75 Pulku Piechoty 1, 41-500, Chorzow, Poland.*

[6]*Institute of Chemistry, University of Silesia, Szkolna 9, 40-006 Katowice, Poland.*

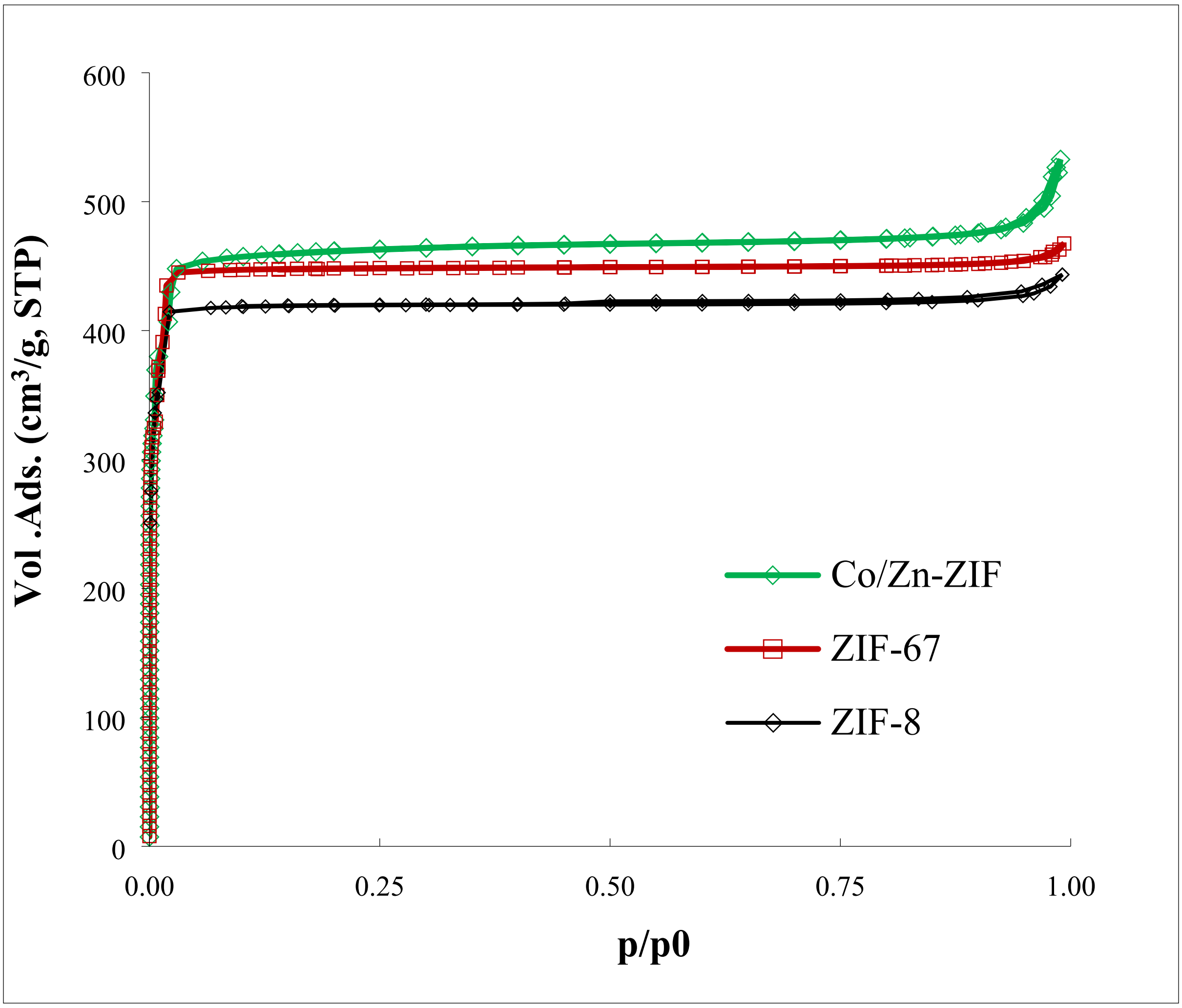

Figure S1. N_2 Isotherms at 77 K of Co/Zn-ZIF (green), ZIF-67 (red) and ZIF-8 (black) materials.

Material	S_{BET}	Micropore Surface area (t-Plot)	Pore Volume	Micropore volume
ZIF-67	1745 m^2/g	1732 m^2/g	0.723 cm^3/g	0.687 cm^3/g
ZIF-8	1648 m^2/g	1602 m^2/g	0.686 cm^3/g	0.643 cm^3/g
Co/Zn-ZIF	1796 m^2/g	1729 m^2/g	0.810 cm^3/g	0.684 cm^3/g

Table S1. Textural values of Co/Zn-ZIF, ZIF-67 and ZIF-8 materials.

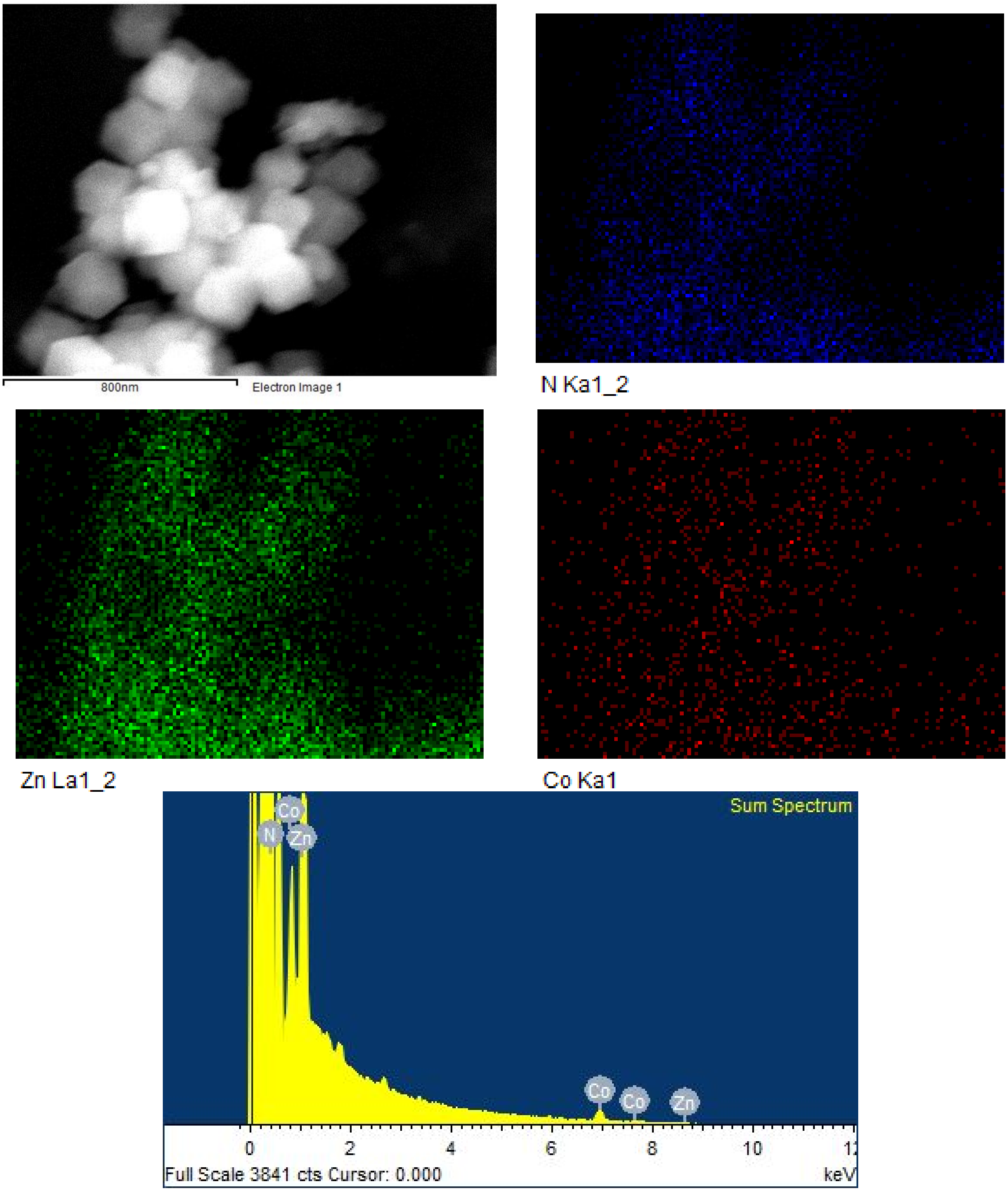

Figure S2. FE-SEM images of bimetallic ($Zn_{0.6}Co_{0.4}$) ZIF and elemental mapping through EDX of the mixed metal frameworks. Elemental mapping of the frameworks using SEM-EDX confirms Zn and Co distribution in the frameworks. The relative concentration of Co and Zn in the frameworks estimated from EDX is $Co_{0.40}Zn_{0.60}$ which shows that Co is incorporated in the frameworks preferentially using FCDS method which is consistent with the previously reported results.[1]

Metallic ions inside hybrid ZIF unit cell

The number of Zn ions per Co ones in the until cell of Co/Zn-ZIF was estimated by the calculation of crystallographic *Z* value defined by the following equation:

$$\boldsymbol{Z} = \boldsymbol{V}(\text{Å}^3) \times \frac{\boldsymbol{\rho}(\boldsymbol{g} \cdot \boldsymbol{cm}^3)}{\mathbf{1.66} \times \boldsymbol{M}\ (\boldsymbol{g} \cdot \boldsymbol{mol}^{-1})} \qquad \text{(eq. 1)}$$

Where *V* is the volume of the unit cell, ρ is the crystallographic density of the compound and *M* is the molar mass of the atoms in the formula.

XRD refinements

Refinements of the XRD patterns of pristine samples of ZIF-67, ZIF-8 and Co/Zn-ZIF were carried out for a deeper study of all frameworks. Due to the asymmetry of the 110 reflection and due to the inconsistencies of the intensities with an empty porous framework, the cited reflection was excluded from the refinement.

**Material**	**Rietveld fit**		**LeBail**	
	**a (Å)**	**size(nm)/strain(%%)**	**a (Å)**	**size/strain**
Co/Zn-ZIF	17.0134(3)	76/10	17.0126(2)	74/10
Co/Zn-ZIF no (110)	17.02031(2)	85/9	17.0205(2)	85/11
ZIF-8	--	--	17.0032(6)	87/8
ZIF-8 no (110)	17.0181(4)	98/2	17.0198(3)	93/3
ZIF-67	17.0447(3)	85/--	17.0439(3)	85/--
ZIF-67 no (110)	17.0474(2)	100/--	17.0473(2)	96/--

Table S2. Data summary from refinements

1. Co/Zn-ZIF

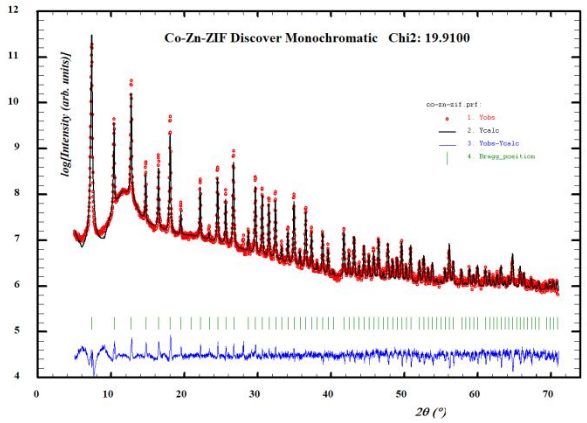

Figure S3. Rietveld refinement to the Co/Zn-ZIF using ZIF-8 structure and 50/50 Co/Zn ratio.

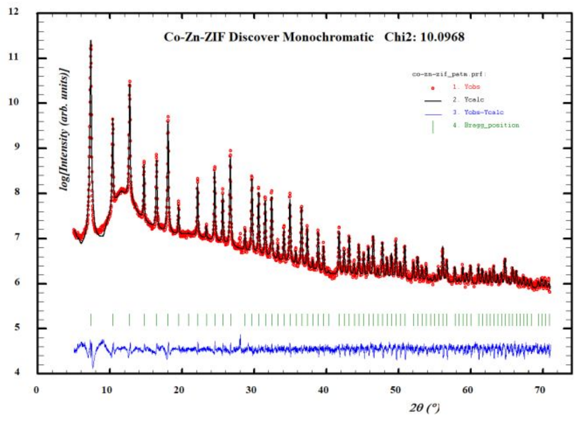

Figure S4. For comparison, the same data fit using only LeBail method.

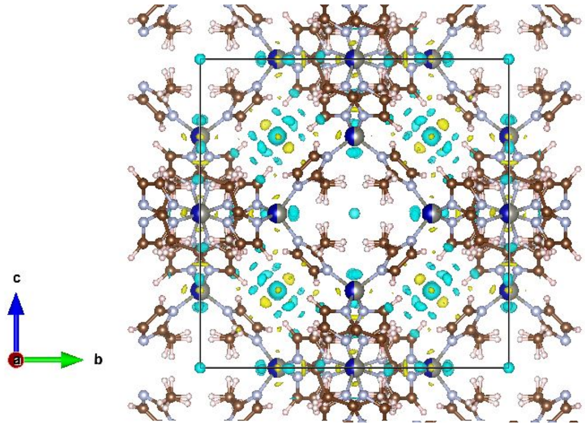

Figure S5. Density difference projected along (100)

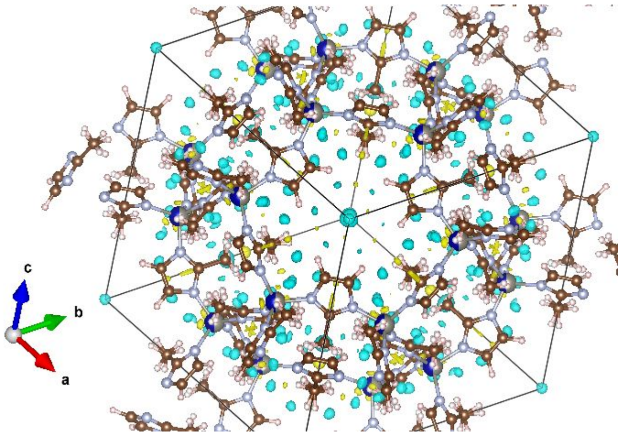

Figure S6. Density difference projected along (111)

These figures suggest that there is something sitting in the middle and in the 6N window

2. ZIF-8

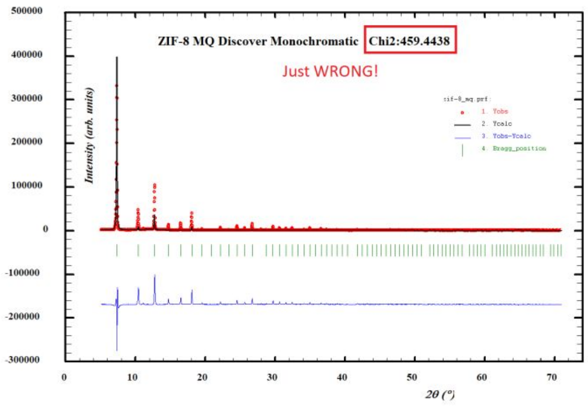

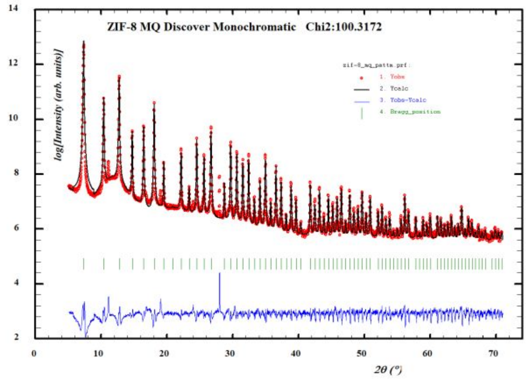

Figure S7. Failed Rietveld refinement since (110) is way above where it should be.

Figure S8. LeBail refinement in the full range.

3. ZIF-67

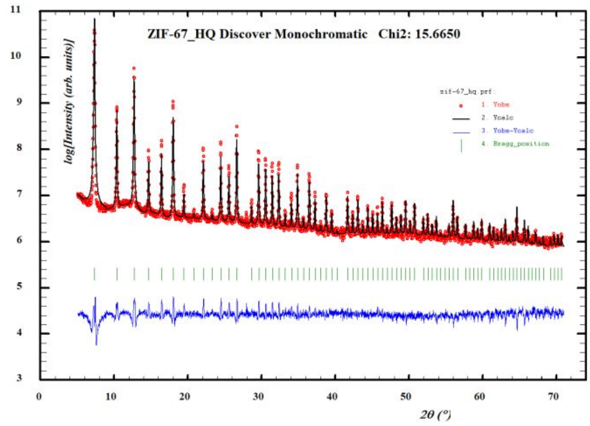

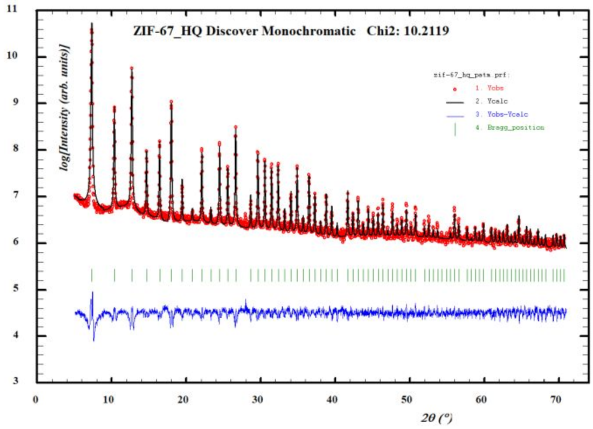

Figure S9. Rietveld refinement of ZIF-67.

Figure S10. LeBail refinement in the full range.

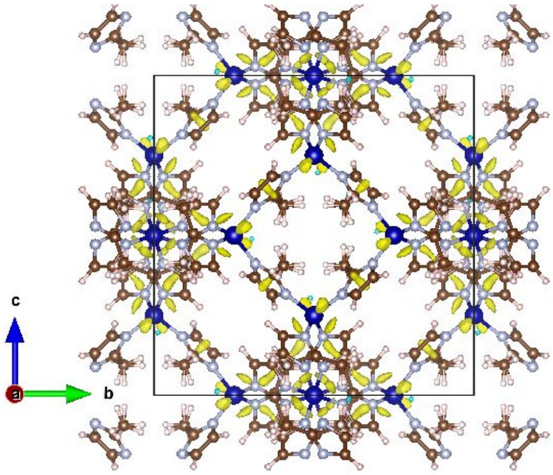

Figure S11. Fourier difference from Figure S10. Interestingly, the Fourier difference for the ZIF-67 is in a different place than in Co/Zn-ZIF.

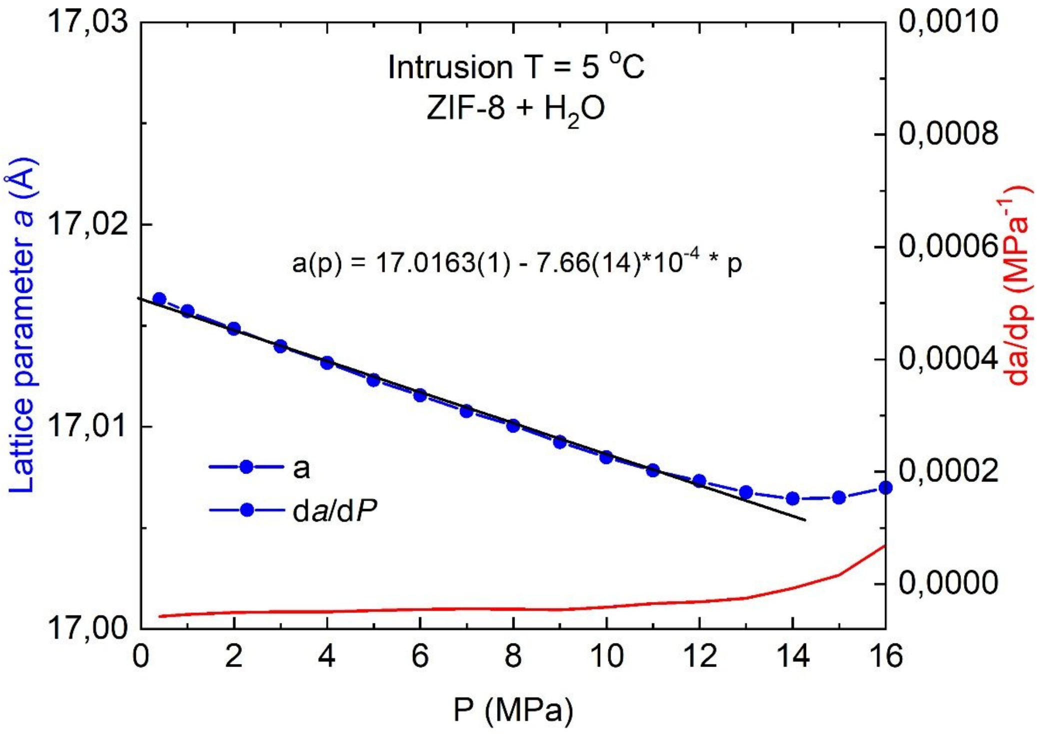

Figure S12. Evolution of a lattice parameter of ZIF-8 during hydrostatic compression. Data were collected at beamline 17-BM at the Advanced Photon Source, Argonne National Laboratory

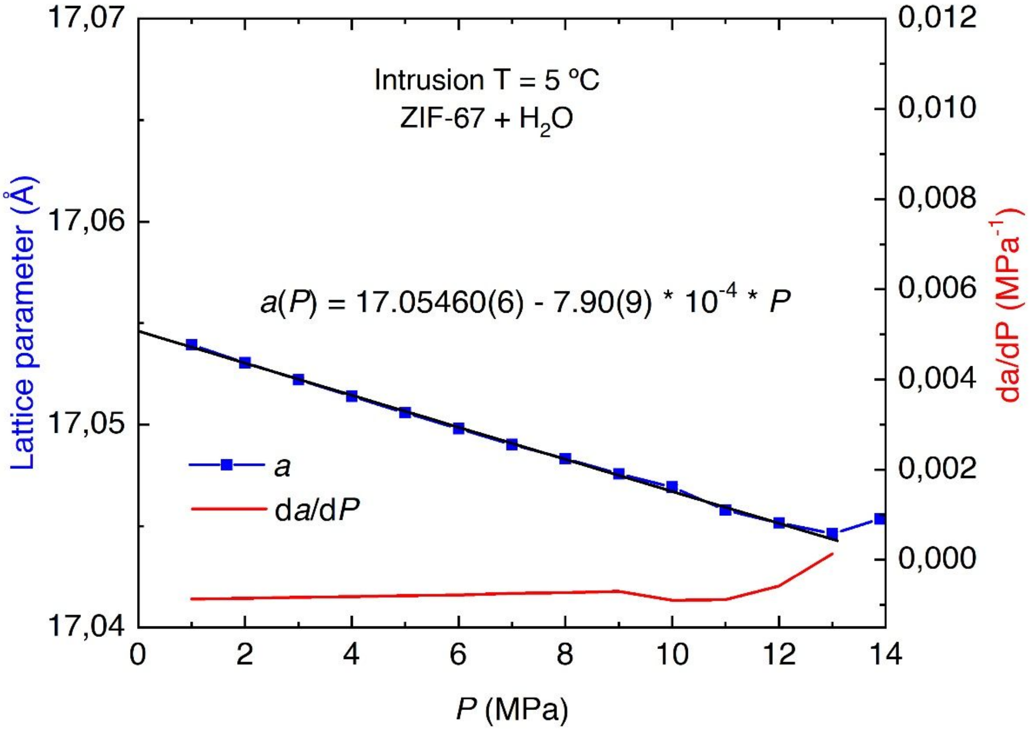

Figure S13. Evolution of a lattice parameter of ZIF-8during hydrostatic compression. Data were collected at beamline 17-BM at the Advanced Photon Source, Argonne National Laboratory. Based on the onset of P_{int} of each ZIF, the relative change of the lattice parameter is 0.038 Å which is 0.002 Δ*a*/*a*. Difference in 1/*a* d*a*/d*P* is (7.90/17.05 - 7.66/17.01) is about 0.013, so in relative terms 2.8%. Around one order of magnitude higher.

Intrusion/extrusion volume - V_{int}/V_{ext} (cm^3/g)

Material/ cycle	1st	2nd	3rd	4th	5th	6th	7th
ZIF-67	0.342/0.335	0.327/0.316	0.302/0.302	0.29/0.29	0.266/0.258	0.253/0.253	0.235/0.231
ZIF-8	0.31/0.30	0.309/0.304	0.302/0.301	0.306/0.301	0.303/0.299	0.293/0.296	0.294/0.294
Co/Zn-ZIF	0.343/0.338	0.342/0.331	0,347 /0.333	0.34/0.336	0.332/0.328	0.339/0.329	0.335/0.327

Table S3. Intrusion/extrusion volume values for ZIF-67, ZIF-8 and Co/Zn-ZIF in all intrusion-extrusion cycles.

(1) Zhou, K.; Mousavi, B.; Luo, Z.; Phatanasri, S.; Chaemchuen, S.; Verpoort, F. Characterization and Properties of Zn/Co Zeolitic Imidazolate Frameworks vs. ZIF-8 and ZIF-67. *J Mater Chem A Mater* **2017**, *5* (3), 952–957. https://doi.org/10.1039/C6TA07860E.
